# A Case of Mycoplasma Infection with an Atypical Presentation of Abducens Nerve Palsy, Erythema Multiforme and Polyarthritis without Respiratory Manifestations

**DOI:** 10.3390/medicina60010036

**Published:** 2023-12-25

**Authors:** Kiyomi Yoshimoto, Masaki Matsubara, Tadanao Kobayashi, Kenji Nishio

**Affiliations:** 1 Department of General Medicine, Nara Medical University Hospital, Kashihara 634-8522, Nara, Japan; m.matsubara@naramed-u.ac.jp (M.M.); nao.k.1115@gmail.com (T.K.); knishio@naramed-u.ac.jp (K.N.); 2 Department of General Medicine, Uda City Hospital, Uda 633-0298, Nara, Japan

**Keywords:** Mycoplasma infection, abducens nerve palsy, erythema multiforme, polyarthritis

## Abstract

*Mycoplasma pneumoniae* is a self-propagating microorganism that commonly causes respiratory tract infections. It can also cause a variety of extrapulmonary symptoms with or independently of respiratory symptoms, such as skin lesions, arthralgia, myalgia, hemolysis, cardiac lesions, gastrointestinal symptoms, and central nervous system lesions, which are rare manifestations reported in approximately 0.1% of cases. In this study, we present a unique case of Mycoplasma-related abducens nerve palsy, polyarthritis, and erythema multiforme without respiratory disease. The patient was a 69-year-old woman who presented to our hospital with a skin rash, fever, arthralgia, and diplopia without respiratory symptoms. Brain magnetic resonance imaging showed optic neuritis on the right side, suggesting the diplopia was caused by right abducens nerve palsy. However, the etiologies of abducens nerve palsy were not revealed by the physical examination, blood biochemistry tests, or bacteriological examinations, including the cerebrospinal fluid examination obtained at admission. Mycoplasma infection was suspected from erythema multiforme revealed by a skin biopsy and polyarthralgia, and it was finally diagnosed according to elevated Mycoplasma particle agglutination (PA) antibodies in paired serum. Though minocycline did not improve her diplopia, the daily administration of 30 mg of prednisolone gradually improved her symptoms, and the Mycoplasma PA antibody titer, which was regularly measured in the clinical course, also decreased, suggesting a relationship between Mycoplasma infection and abducens nerve palsy. This is the first case of isolated abducens nerve palsy, which was reported as the only central neurological symptom in an adult patient with Mycoplasma infection. The mechanism or pathogenesis of CNS manifestations caused by *Mycoplasma pneumoniae* remains to be elucidated, and further investigation is needed. Hence, Mycoplasma infection is a common disease. Clinicians should be aware of the diverse manifestations, including abducens nerve palsy, of Mycoplasma infection and should consider Mycoplasma infection even in the absence of typical respiratory symptoms.

## 1. Introduction

*Mycoplasma pneumoniae*, primarily recognized as a respiratory pathogen, causes upper respiratory tract infections and community-acquired pneumonia. It is also implicated in triggering asthma exacerbations [1,2]. Studies have reported that up to 25% of patients with Mycoplasma-induced respiratory tract infections exhibit extrapulmonary lesions [3]. These manifestations can include, but are not limited to, skin lesions, arthralgia, myalgia, hemolysis, cardiac and gastrointestinal symptoms, as well as neurological complications. Neurological lesions, which are not always accompanied by respiratory symptoms, are challenging to diagnose. Among these, central nervous system (CNS) involvement by *Mycoplasma pneumoniae*, presenting as encephalitis, meningitis, peripheral neuropathy, transverse myelitis, acute disseminated encephalomyelitis (ADEM), Guillain–Barre syndrome, and cranial nerve palsy, is serious and prognostically significant, occurring in approximately 0.1% of all patients [4]. This report details an unusual case of abducens nerve palsy in a patient without respiratory symptoms caused by Mycoplasma infection. The patient, who also presented with fever, erythema multiforme, and polyarthritis, showed improvement following corticosteroid treatment, underscoring the importance of accurate diagnosis and management of such cases.

## 2. Case Presentation

**Figure 1:**
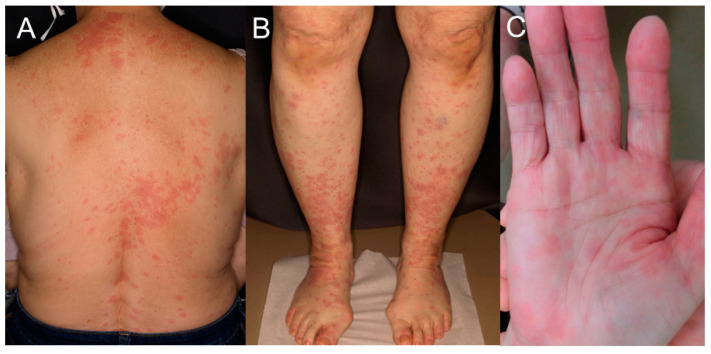
Erythema of the back (**A**), front of the lower legs (**B**), and right palm (**C**) at onset.

**Figure 2:**
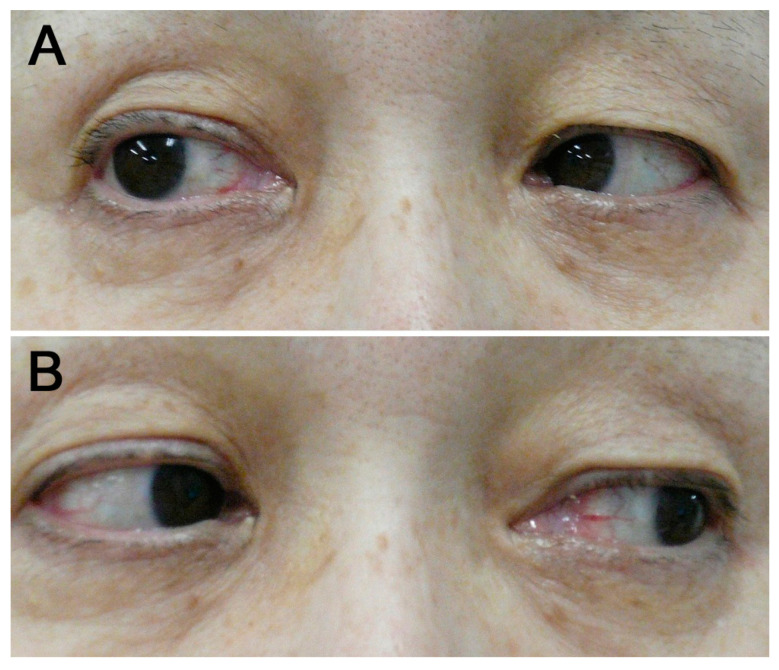
(**A**) Unsuccessful abduction of the right eye during right gaze. (**B**) Normal adduction of right eye during left gaze.

The patient was a 69-year-old woman. She had a history of urinary tract stones, an operation for endometriosis, herpes zoster, and glaucoma. A skin rash appeared on her right upper limb, which gradually spread to her whole body 9 days before admission. She had a fever 3 days before admission, and arthralgia and diplopia were observed 1 day before admission; thus, she was referred to our department. As for her vital signs, her blood pressure was 115/66 mmHg, her heart rate was 87 bpm, her blood temperature was 37.4 °C, her respiratory rate was 20/min, and her blood oxygen saturation was 96% (room air). A physical examination revealed erythema with pruritus all over her body (Figure 1), arthritis of both hands, elbows, knees, and ankles, and swollen lymph nodes in the right inguinal region. Neurological examination revealed limited right ocular abduction (Figure 2), but no other abnormal neurological findings, including decreased tendon reflexes, were obtained. Hess chart was performed, and diplopia was identified. Her laboratory data are shown in Table 1.

**Figure 3:**
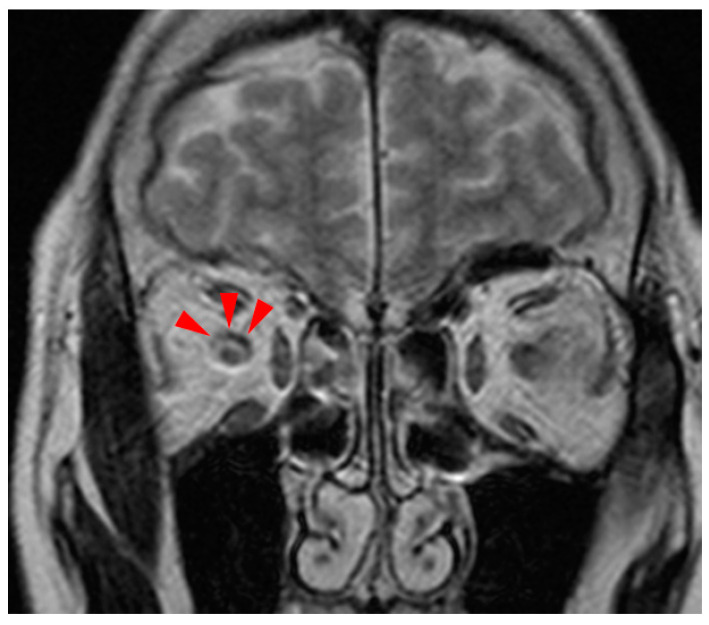
Magnetic resonance imaging T2-weighted image shows high signal in the right optic nerve sheath (red arrows), suggesting right optic perineuritis.

A cerebrospinal fluid (CSF) examination showed no abnormalities, and two sets of blood cultures showed no evidence of bacterial growth. A CT scan of the chest showed no evidence of pneumonia, and a magnetic resonance imaging (MRI) scan of the head showed a high signal of the right optic nerve sheath on T2-weighted images, suggesting right optic nerve perineuritis (Figure 3).

**Figure 4:**
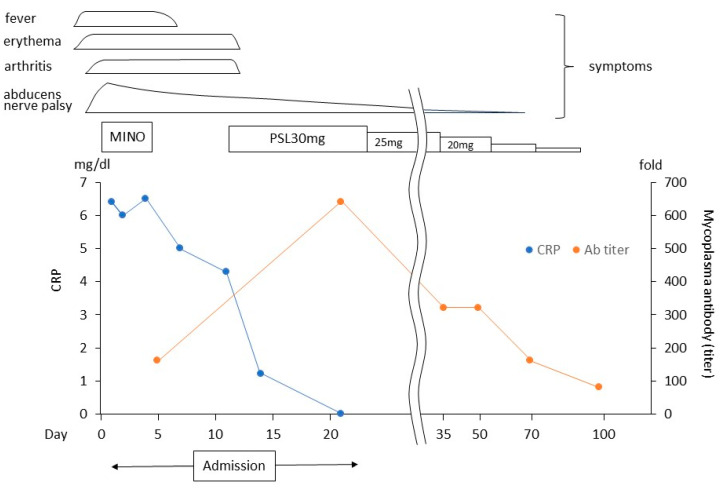
Clinical course of the patient. MINO, minocycline; PSL, prednisolone; CRP, C-reactive protein.

She was admitted to our hospital, and treatment with minocycline 200 mg/day was started on suspicion of Mycoplasma infection. However, symptoms such as erythema, arthritis, and abducens nerve palsy did not improve, and serum C-reactive protein (CRP) levels did not decrease; thus, the administration of minocycline was discontinued after 5 days of use. A skin biopsy was performed on admission day, and it was revealed that the pathological findings were consistent with erythema exudative multiforme. The fever resolved on day 7, but the other symptoms described above did not improve. A right inguinal lymph node biopsy was performed on day 11 to determine the cause of the fever up, elevated blood levels of CRP, and soluble interleukin-2 receptor, and it turned out that dermatopathic lymphadenopathy was likely, and there was no evidence of malignancies or epithelioid cell granuloma common in sarcoidosis. Prednisolone (PSL) 30 mg/day was started on day 12, and soon, erythema and arthritis resolved, and the abducens nerve palsy also showed gradual improvement. The patient was discharged on day 22. Subsequently, PSL was gradually tapered and successfully discontinued, with no recurrence of abducens nerve palsy observed. The Mycoplasma particle agglutination (PA) antibody titer demonstrated a progression from 160-fold on day 5 to 640-fold on day 21, then decreased to 320-fold on day 35, and to 80-fold by day 97. This trend parallels the resolution of abducens nerve palsy. In conjunction with the concurrent skin rash and arthralgia, these observations support a diagnosis of acute Mycoplasma infection, potentially linked to the abducens nerve palsy. The clinical course of the patient is shown in Figure 4.

## 3. Discussion

This paper describes a case of *Mycoplasma pneumoniae* infection presenting with abducens nerve palsy, fever, erythema multiforme, and polyarthritis in the absence of respiratory symptoms. The patient’s condition improved following corticosteroid treatment, not minocycline. Diagnosing Mycoplasma infection in patients with neurological symptoms but without respiratory manifestations can be challenging. In this case, the specific characteristics of the skin rash (erythema multiforme exudativum) and concurrent polyarthritis raised the suspicion of Mycoplasma infection. This led to the initiation of minocycline treatment and the measurement of Mycoplasma antibody titers at admission. However, a definitive diagnosis of Mycoplasma infection relies on comparing antibody titers in both the acute and convalescent phases. The differential diagnosis for this patient initially focused on three symptoms: abducens nerve palsy, polyarthritis, and erythema multiforme.

A wide variety of etiologies are known for abducens nerve palsy, including cerebral infarction, microvascular, aneurysm, neoplastic, meningitis, and Fisher syndrome [5,6], all of which were ruled out by the physical examination, blood biochemistry tests, bacteriological examinations, and imaging tests in this patient. Since cerebral MRI suggested right optic nerve perineuritis, we considered the notion that abducens nerve palsy was caused by orbititis, which could be induced by systemic inflammatory disease or infectious disease in addition to idiopathic [7]. Neurological consultation suggested the possibility of mononeuritis from collagen disease, antineutrophil cytoplasmic antibodies (ANCA)-associated vasculitis, or sarcoidosis. Despite an elevated antinuclear antibody titer of 1:160, the absence of symptoms like stomatitis, photodermatosis, pleuritis, or pericarditis, and the lack of specific laboratory markers (a mildly elevated blood sedimentation rate but no decrease in lymphocyte count or complement), made systemic lupus erythematosus (SLE) an unlikely diagnosis. Additionally, the normal levels of myeloperoxidase–antineutrophil(MPO)-ANCA, proteinase 3(PR3)–ANCA, and other specific autoantibodies, including anti-CCP, anti-RNP, and anti-SS-A/B antibodies, reduced the likelihood of ANCA-associated vasculitis or collagen diseases. The pathological findings of the lymph node biopsy were not suggestive of sarcoidosis. Based on these results, the etiology of the abducens nerve palsy of the patient was considered due to infectious disease. In children, abducens nerve palsy is rarely caused after an upper respiratory tract infection known as “benign isolated abducens nerve palsy”, and it has been suggested to be related to Mycoplasma or other viral infections [8,9]. In adults, we only found a case of a 52-year-old man who developed abducens nerve palsy due to a Mycoplasma infection, showing choreoathetoid movements and an acute psychosis, suggesting he had suffered from encephalitis and not exclusively abducens nerve palsy [10]. In the current case, the abducens nerve palsy improved as the Mycoplasma antibody titer decreased, indicating a relationship between Mycoplasma infection and abducens nerve palsy. Therefore, this is the first case of Mycoplasma infection that only shows abducens nerve palsy as the CNS symptom in an adult patient.

Polyarthritis accompanied by fever can be attributed to various causes, including infectious arthritis, collagen diseases such as rheumatoid arthritis, Still’s disease, and SLE, reactive arthritis and crystal-induced arthritis [11]. In the case under discussion, collagen diseases were deemed unlikely due to the absence of autoantibodies and only mildly elevated ferritin levels, and crystal-induced arthritis was considered improbable as it is rarely associated with erythema multiforme. When diagnosing polyarthritis, the key symptoms or features are as follows: fever preceding arthritis, migratory arthritis, symmetric small-joint synovitis, and leukocytosis or leukopenia [11]. In polyarticular infection, Neisseria Species, *Streptococcus pneumoniae, Haemophilus influenzae, group G streptococci*, and *Mycoplasma pneumoniae* are known to cause polyarthritis rather than monoarthritis. In the present case, fever preceded the onset of polyarthritis, the large joints were painful and non-migratory, and a normal white blood cell count led to the consideration of Mycoplasma infection as a potential cause of polyarthritis at the time of admission.

Though erythema multiforme could be caused by a variety of factors, such as infection, medication, malignancy, and collagen diseases, infections are the most common cause [12]. Herpes simplex virus (HSV) is most commonly identified, and *Mycoplasma pneumoniae* is also a significant contributor. In this case, an HSV infection was needed to be ruled out, prompting an HSV antibody test. The results revealed a negative anti-HSV IgM antibody and a positive anti-HSV IgG antibody, indicating a prior HSV infection. Together with the polyarthritis described above, we suspected the possibility of Mycoplasma infection from the beginning.

In the current adult case, only abducens nerve palsy was the CNS symptom caused by Mycoplasma infection. Other than the abducens nerve among cranial nerves impaired due to Mycoplasma infection, we could detect a child case of oculomotor nerve palsy [13] and facial nerve paralysis in some adult patients [14]. CNS involvement induced by *Mycoplasma pneumoniae* is the most difficult situation to be diagnosed and treated since the diagnosis is challenging and the prognosis is sometimes poor. Neurological manifestations of *Mycoplasma pneumoniae* infections are thought to arise from three distinct mechanisms [15]. The first, a direct mechanism, involves damage to nervous tissue directly attributable to the local activity of *Mycoplasma pneumoniae*. The second, an indirect mechanism, is primarily autoimmune. The third is a vascular mechanism, characterized by local vasculitis or thrombotic vascular occlusion, which may result from either direct or indirect pathways [16]. These mechanisms are not mutually exclusive, which may explain the detection of lesions with varied pathogens in certain patients These mechanisms are not mutually exclusive, which may be explained by the detection of lesions with varied pathogeneses in certain patients. [3]. In the presented case, the likelihood of abducens nerve palsy resulting from direct Mycoplasma infiltration appears low. Typically, if cranial nerves are directly infiltrated, spinal fluid tests would reveal abnormalities, such as elevated cell counts and protein concentrations. However, in this instance, no such abnormalities were observed in the cerebrospinal fluid. Furthermore, the patient’s lack of improvement following minocycline treatment suggests an alternative cause. In Japan, minocycline is recognized as effective against Mycoplasma infections [17]. Hence, in cases of direct infiltration, minocycline treatment would be expected to alleviate symptoms of abducens nerve palsy, albeit mildly. Notably, surveillance data from Japan indicates a rise in macrolide-resistant Mycoplasma strains compared to Western regions [18,19,20], warranting the administration of minocycline in this case. Neuropathies manifesting over eight days post-Mycoplasma infection are classified as late-onset diseases [15]. In these cases, unlike some early onset diseases, antibodies to *Mycoplasma pneumoniae* can be detected in CSF and/or serum in a significant number of cases, suggesting that late-onset disease is primarily based on autoimmunity. Furthermore, since the symptoms were alleviated and disappeared with corticosteroids, we believe that the abducens nerve palsy in this case may have been caused by an immune mechanism. Additionally, the possibility that the abducens nerve palsy was induced by a vasculitis-like mechanism cannot be discounted. Though several studies tried to reveal the mechanism of CNS manifestations caused by *Mycoplasma pneumoniae* [3,16,21], they could not achieve sufficient results, and the pathogenesis of *Mycoplasma pneumoniae*-induced complications of CNS still remained to be elucidated. A major hurdle in this area is the difficulty in establishing a direct causal link between Mycoplasma infection and CNS symptoms. Moreover, the absence of respiratory symptoms in some patients with CNS manifestations may lead to an oversight of Mycoplasma infection, complicating the diagnostic process. Furthermore, the presence of asymptomatic carriers adds another layer of complexity [22].

As for the diagnosis of *Mycoplasma pneumoniae*, available diagnostic assays have some limitations, such as restricted use, lack of validation, and long turnaround times. Though isolation and culture are the gold standard methods, serological methods, the loop-mediated isothermal amplification (LAMP) method, or a rapid antigen test are widely used in routine care since the isolation culture method requires a special culture medium and the proliferation rate of *Mycoplasma pneumoniae* is low compared to other bacteria [23]. Hence, our diagnosis is based on elevated Mycoplasma PA antibodies in paired serum. We regularly repeated measuring Mycoplasma PA antibodies after discharge, considering the possibility of a false positive or cross-reaction. In fact, the PA antibody titer gradually decreased without reascending, suggesting accuracy of the PA antibody in the current patient.

## 4. Conclusions

Herein, we report the first case of Mycoplasma infection, which only shows abducens nerve palsy as the CNS symptom, showing a variety of extrapulmonary symptoms without respiratory manifestations in an adult patient. We suggest that a meticulous examination and diagnostic approach are crucial in identifying Mycoplasma infection. Long-term monitoring of Mycoplasma PA antibodies, as observed in this case, enhances the accuracy of the diagnosis, further supported by the presence of erythema multiforme. Additionally, a comprehensive literature review revealed that this patient’s presentation, with abducens nerve palsy as the sole CNS manifestation of Mycoplasma infection, has not been previously documented. Although Mycoplasma infection is a common disease, it can manifest a diverse array of symptoms, posing diagnostic challenges, as demonstrated in this case. This case illustrates that in patients presenting with neuropathy, including abducens nerve palsy, a Mycoplasma infection should be considered a differential diagnosis, regardless of the presence of respiratory symptoms. Consequently, conducting tests such as Mycoplasma antibody titers is advisable in such scenarios.

## Figures and Tables

**Table 1:** Laboratory data upon admission.

Parameter	Recorded Value	Standard Value
White blood cells (μL)	4100	3300–8600
Stab-formed neutrophils (%)	8.0	0.0–7.0
Segmented neutrophils (%)	73.0	30.0–79.0
Lymphocytes (%)	12.0	9.0–55.0
Monocytes (%)	6.0	0.0–13.0
Eosinophils (%)	0.0	0.0–6.0
Basophils (%)	1.0	0.0–2.0
Red blood cells (10^4^/μL)	409	386–492
Hemoglobin (g/dL)	12.6	11.6–14.8
Platelets (10^4^/μL)	12.0	15.8–34.8
ESR 1 h (mm)	49	3–15
Total protein (g/dL)	6.9	6.6–8.1
Albumin (g/dL)	4.1	4.1–5.1
Total bilirubin (mg/dL)	1.1	0.4–1.5
Aspartate aminotransferase (U/L)	27	13–30
Alanine transaminase (U/L)	35	7–23
Lactate dehydrogenase (U/L)	228	124–222
Gamma glutamyl transpeptidase (U/L)	41	9–32
Alkaline phosphatase (U/L)	177	106–322
Creatine kinase (U/L)	71	41–153
Blood urea nitrogen (mg/dL)	13	8–20
Creatinine (mg/dL)	0.55	0.46–0.79
Sodium (mEq/L)	136	138–145
Potassium (mEq/L)	3.9	3.6–4.8
Chloride (mEq/L)	99	101–108
C-reactive protein (mg/L)	6.4	<0.2
Ferritin (ng/mL)	317.7	3.0–55.0
Soluble interleukin-2 receptor (U/mL)	2210	157–474
Matrix metalloproteinase-3 (ng/mL)	44.4	11.0–54.5
Antinuclear antibody (fold)	160	<40
Rheumatoid factor (U/mL)	28	<15
Anti-CCP antibody (U/mL)	<0.6	<0.6
Anti-AQP4 antibody (U/mL)	<1.3	<5.0
MPO-ANCA (U/mL)	<0.1	<0.1
PR3-ANCA (U/mL)	<0.1	<0.11
Anti-Herpes simplex virus IgM *	(−)	(−)
Anti-Herpes simplex virus IgG *	(+)	(−)
Anti-Varicella zoster virus IgM *	(−)	(−)
Anti-Varicella zoster virus IgG *	(+)	(−)

* EIA; Enzyme Immunoassay. ESR, erythrocyte sedimentation rate; Anti-CCP, anti-cyclic citrullinated peptide; Anti-AQP4, Anti-Aquaporin 4; PR3-ANCA, proteinase 3–antineutrophil cytoplasmic antibodies; MPO-ANCA, myeloperoxidase–antineutrophil cytoplasmic antibodies; Ig, immunoglobulin.

## Data Availability

The data presented in this study are available on request from the corresponding author. The data are not publicly available due to privacy restrictions.
